# What Judges Want to Know From Forensic Evaluators in Child Custody and Child Protection Cases: Analyzing Forensic Assignments With Latent Dirichlet Allocation

**DOI:** 10.3389/fpsyg.2021.603597

**Published:** 2021-04-13

**Authors:** Jelena Zumbach, Renate Volbert

**Affiliations:** ^1^Psychologische Hochschule Berlin, Berlin, Germany; ^2^Charité - Universitätsmedizin Berlin, Corporate Member of Freie Universität Berlin and Humboldt-Universität zu Berlin, Institute of Forensic Psychiatry, Berlin, Germany

**Keywords:** forensic evaluation, referral legal question, child custody, child endangerment, latent Dirichlet allocation

## Abstract

This study analyzes the questions on aspects of child custody, visitation rights, or child endangerment that judges pose to forensic psychologists in family law proceedings. Before conducting a psychological evaluation, the legal question in the referral has to be translated into case-specific, forensically relevant issues. The only overarching principle guiding this process is the “best interests of the child” criterion. Literature indicates that judges often struggle to define what variables should be specified for a psychological evaluation in their referral questions. This study aims to contribute to a better understanding of the information judges would like to ascertain from psychological evaluators in child custody and child protection proceedings—an understanding allowing a clearer determination of whether forensic psychologists as experts can deliver this information. Latent Dirichlet allocation (LDA) is used to analyze the referral questions that these judges pose to forensic evaluators in terms of (a) underlying topics (latent dimensions) that can be identified within the referral questions and (b) the probability distributions of legal terms and forensic issues contained in the referral questions. This analysis is based on unclassified text data extracted from German court files. Five topics (latent dimensions) were identified within referral questions resembling cases when the issue was as follows: (a) potential child endangerment in the context of visitation contacts, (b) a possibly limited parenting capacity and its potential effects on child well-being, (c) an impairment of the child has already occurred or could occur, (d) a better option concerning custody and residence, and (e) an unclear topic addressing questions on custody, residence, and visitation in which no specific psychological constructs are involved. In four of the five topics, judges utilize their referral questions to ask for case-group-specific psychological information. In one topic addressing questions on custody, residence, and visitation, judges seem to struggle to define criteria that forensic evaluators should assess. Overall, results help to identify and define more clearly the relevant constructs that forensic experts should examine from the perspective of the courts with the goal of making clearer and more accurate recommendations.

## Introduction

During a criminal or civil trial, a forensic evaluation is conducted at the interface between psychological/psychiatric and legal expertise. It can take place within a variety of legal settings, in both criminal and civil law (see the *Specialty Guidelines for Forensic Psychology* from the American Psychological Association, 2012). This study focuses on the questions that judges ask about child custody, visitation rights, or child endangerment when assigning evaluations to forensic psychologists in family law proceedings.

When analyzing referral questions and forensic assessments, it is useful to distinguish between the referral *legal question* and the *relevant forensic issues* that are associated with it. The referral *legal question* is defined as “the ultimate issue to be decided by the court” (Heilbrun et al., 2009, pp. 100–101; LaDuke, 2017, p. 160). *Relevant forensic issues* are defined as “capacities and abilities that are part of the ultimate legal question and can be measured by forensic evaluators” (Heilbrun et al., 2009, pp. 100–101; LaDuke, 2017, p. 160). Relevant forensic issues are often referred to as psycholegal issues and comprise questions on case-relevant psychological constructs (Heilbrun et al., 2009; LaDuke, 2017).

For example, in family law proceedings, the ultimate legal issue on which the court has to decide may be the child's custody. Forensic psychologists may provide data and expertise on a number of forensic issues such as the parenting capacity, the mental health of parents and children, and their relationships and attachments. Identifying the forensic issues has significant ethical and practical implications, because it guides the conceptualization, conduct, and communication of the forensic evaluation (LaDuke, 2017). The translation of the referral legal question into case-specific forensic relevant issues is therefore a key variable in assuring that forensic evaluations are conducted accurately and effectively (American Psychological Association, 2013; LaDuke, 2017).

The process of translating the referral legal question into forensic issues may be the responsibility of the forensic psychologist. Similarly, the judge may already indicate forensic issues and tasks on case-relevant constructs to the forensic evaluator within the referral question. This creates a certain margin of shared responsibility between judge and forensic evaluator at this psycholegal interface. This applies at least to civil law systems such as Germany in which the judge has a more active and managing role in the process and is tasked with investigative functions.

### The Role of Experts in Civil Law vs. Common Law Systems

In civil law systems, the decision to appoint an expert is taken by the court. The courts select the expert and notify the parties of such a decision. The expert is an “auxiliary” commissioned by the court (i.e., an assistant, the German term is “*Gehilfe des Gerichts*”). Experts operate under the court's direction and have a special status. They must limit themselves to the scope of research that the court has specifically commissioned them to perform, typically through a set of questions addressed to the expert. The court, however, is not bound by the expert opinion. In principle, courts can disregard the expert opinion if they do not find it convincing (see World Bank, 2010).

In some common law systems such as those of the United States, the law also provides explicitly for court-appointed experts; however, these are relatively rare in the practice of litigation. Generally, in common law systems, the judge is more of a passive and neutral arbiter who listens to what the parties bring forward, including contradictory expert opinions. In these systems, experts function as witnesses for the respective parties who hire (and pay) them. In common law countries, the court acts as a gatekeeper for admitting scientific knowledge into court proceedings (see World Bank, 2010). The definition of this gatekeeper role goes back mainly to the U.S. Supreme Court decision in *Daubert v. Merrell Dow Pharmaceuticals, Inc*., 509 U.S. 579 (1993), in which the court decided that the expert's testimony should be based on scientific knowledge (Daubert, at 590) and that “the trial judge must ensure that any and all scientific testimony or evidence admitted is not only relevant, but also reliable” (Daubert, 1993).

In both common law and civil law systems, an expert opinion should cover “issues of fact” and may not substitute or take the place of the court opinion by offering “legal” conclusions (i.e., whether the conduct of a party is “legal or illegal”; see World Bank, 2010). This provides ground for a discussion that forensic assignments containing legal questions or referencing judicial statutes are wrongful and highly problematic, because they may lead the forensic expert to offer legal conclusions by answering such referral questions (see Coester, 2016; Bergmann, 2018).

### Problematic Assignments in Family Law Practice

Assignments that comprise broad normative questions (e.g., “Which custody or visitation regulations serve a child's best interest?” and “Is there a risk to the well-being of the child?” see Jameson et al., 1997; Gould, 1999) are considered to be problematic. Assignments that simply reference judicial statutes are also discussed as highly problematic and wrongful (e.g., “Conduct an evaluation according to Code § [*citation*]”; Coester, 2016, p. 579; see also Bergmann, 2018). Some authors argue that the forensic assignment must not contain any reference to legal statutes and terms, because a mental health professional is not qualified to address legal issues (Bergmann, 2018).

On the other hand, leaving the process of translating legal questions into case-specific psychological-forensic issues fully in the judge's hands may outreach her or his boundaries as a legal professional (Jameson et al., 1997). Literature from both Germany and the United States indicates that judges often struggle with defining what variables should be specified for psychological evaluation in family law (Gould, 1999; Krauss and Sales, 1999, 2000; Grisso, 2005; Bergmann, 2018; Melton et al., 2018).

To the best of our knowledge, data have yet to be published on the referral questions (German) judges pose to forensic evaluators in family law matters. This is especially problematic in family law, because the variety of legal questions that may lead to a psychological evaluation is particularly broad. We can identify at least four groups of legal questions for which psychological evaluations are frequently commissioned in Germany. Those involve (a) child custody after parental separation that typically address where the child should live; (b) visitation with a non-resident parent that typically occurs after parental separation when the child's residence is not questioned; (c) questions on both child custody and visitation that typically occur after parental separation when both the child's residence and visitation with the non-resident parent are being questioned; and (d) questions on child endangerment and child protection.

Therefore, the number of forensic issues that potentially may be assessed is equally broad. For example, issues can focus on aspects of parents such as their mental health and its implications for their parenting capacity, intimate partner violence allegations, or parental conflict and cooperation. Child-related issues may include the psychological adaptation and functioning of the child, alienation, the child's wishes and preference, and the child's relationships with and attachment to the parents (American Psychological Association, 2010, 2013; Arbeitsgruppe Familienrechtliche Gutachten, 2019).

### Forensic Assignments and the Best Interests of the Child Principle

The only overarching principle that guides decision making in the context of child custody and parental rights is the *best interests of the child* principle. This is a legal principle that derives from the United Nations Convention on the Rights of the Child (UNCRC). It calls for the determination of a variety of factors that best suit the child's needs in a specific situation (Miller, 2002). The principle emphasizes the importance that cases should be decided with the child's interest foremost and not that of the parents. What is in the child's best interest, however, often becomes a matter of contention (Ladd, 2017).

Jurisdictions generally do not have statutory provisions explicitly defining what is meant by the “best interests of the child.” As highlighted by the Commission on European Family Law, the best interest of the child is a notion whose content cannot be fixed permanently but must reflect the prevailing values in the society in question and the concrete situation of the specific child (Boele-Woelki et al., 2007). The term generally refers to the deliberation that courts undertake when deciding what type of services, actions, and orders will best serve a child as well as who is best suited to take care of that child. However, child and family psychology may influence developing jurisprudence. Certain goals stipulated by the law may be interpreted as a definition of what is meant by the child's best interests, such as the child's right to care, protection, a good upbringing, and increasing autonomy with growing maturity (Boele-Woelki et al., 2007).

Therefore, the child's best interests are usually operationalized by a number of factors related to the child's circumstances along with the parent or caregiver's circumstances and capacity to parent, with the child's ultimate safety and well-being taken as the paramount concern. Factors listed in different instruments may vary considerably both nationally and internationally (see Gould, 1999; Child Welfare Information Gateway, 2016; Coester, 2016; Gould et al., 2016; Bergmann, 2018). Commonly named factors in both national and international literature (see Child Welfare Information Gateway, 2016; Dettenborn and Walter, 2016; Kindler, 2018) include the following:

- relationship and attachment between the child and caregivers;
- mental and physical health needs of the child as well as physical, emotional, or behavior problems of the child;
- the child's need for stability and continuity;
- the voice/preference/wishes of the child;
- the parenting capacity, parenting skills, and educational skills;
- the mental and physical health of the parents.

Practical experience has shown that judges frequently refer to these criteria when formulating their forensic assignments. However, given that there are various legal and psychological definitions of the child's best interest criterion, there is no clear consensus defining the dimensions and variables to be evaluated when determining the child's best interests either within Germany (Coester, 2016; Bergmann, 2018) or internationally (Gould, 1999; Gould et al., 2016). Therefore, the clarity and scope of concepts that guide custody evaluations remain somewhat poorly defined.

### Aims of This Study

As a result, the sufficiency and relevance of the evaluation itself, as well as the potential for misuse of influence by the forensic evaluators, have been the subject of a critical debate (Krauss and Sales, 1999, 2000; Grisso, 2005; Martindale and Gould, 2007; Bergmann, 2018; Melton et al., 2018). Given the high practical relevance of the forensic assignment as well as the highlighted research gaps, the aim of this study was to clarify the identification and definition of the relevant constructs to be examined by forensic experts from the perspective of the courts. This helps to provide clarity on what is actually at stake within the questions posed by the courts and to better understand current procedures at this psycholegal interface in family law.

Therefore, we analyzed the referral questions that judges pose to forensic evaluators in family law proceedings with regard to (a) underlying topics (latent dimensions) that can be identified within the referral questions and (b) the probability distributions of legal terms and forensic issues within them. A better understanding of what judges would like to ascertain from psychological evaluators in child custody and child protection proceedings will allow us to identify and define more clearly the relevant constructs that forensic experts need to examine from the perspective of the courts. This can help provide clarity on what is actually at stake within the questions posed by the courts and whether that can be fulfilled by forensic psychologists as experts.

Our analysis was based on unclassified text data extracted from German court files. In an exploratory approach, we used latent Dirichlet allocation (LDA) to identify topics (latent dimensions) and probability distributions among referral questions. To the best of our knowledge, this is the first study to employ the LDA method to discover topics and identify latent structures by analyzing raw data derived from court files.

## Methods

### Sample

The sample consisted of 196 forensic assignments (text corpora in exact wording) by family court judges to forensic evaluators affiliated with a forensic practice in Northern Germany. All assignments in family law proceedings directed to the institution in 2018 and 2019 were included in the data collection process (i.e., issues regarding child custody, visitation, termination of parental rights, and reinstatement of parental rights after an earlier placement in foster care). Forensic assignments from 24 different courts in Northern Germany were included in the database. The individual assignments addressed a range of one to six children within each family.

### Data Analysis

We applied topic modeling using LDA. Latent Dirichlet allocation is an unsupervised machine learning topic model developed by Blei et al. (2003) that allows for the automatic clustering of any kind of text documents (text corpora) into a chosen number of clusters of similar content, referred to as topics. In its clustering, LDA makes use of a probabilistic model of the text data: co-occurrences of words are used by LDA to describe each topic as a probability distribution over words and each document as a probability distribution over topics. Latent Dirichlet allocation detects previously hidden relationships between documents and topics (Blei et al., 2003).

So far, topic analysis has been studied in such areas as online health communities (see Nguyen et al., 2015), sustainable development (see Mora et al., 2018), and customer review (see Mou et al., 2019). Most of the data examined were social media or company feedback posts (Nguyen et al., 2015; Mou et al., 2019) and titles, abstracts, or full texts of scientific studies (Mora et al., 2018). To the best of our knowledge, this is the first study to use topic modeling to analyze judicial documents.

#### Description of the Latent Dirichlet Allocation Method

In the dataset, each observation represented one of the documents as text strings. Each text string consisted of the full (set of) question(s) that a judge posed to the evaluator per case. Each document therefore constituted the entire single forensic assignment in exact wording.

The LDA consists of two parts: The first part determines the probabilistic model that describes the text data as a likelihood function. The underlying principle of the LDA model is its allocation of words from multiple, separate reviews to new documents, and assigning a probability to each word. Consequently, each new document represents a topic consisting of some highly related co-occurring words.

In the second part, LDA classification is based on finding the optimal topic assignment for each word in each document, and the optimal word probabilities for each topic that maximizes this likelihood. For this analysis, we followed suggestions in the literature and used an approximate inference algorithm developed by Griffiths and Steyvers (2004): the Gibbs sampling method. Gibbs sampling is a Markov chain Monte Carlo algorithm based on repeatedly drawing new samples conditional on all other data. As a Bayesian technique, the Gibbs sampler relies on iteratively updating the topic assignment of words conditional on the topic assignment of all other words (see Schwarz, 2018).

#### Data Analytic Procedure

The LDA analysis first splits each document into individual words that are then randomly assigned to one of the *T* topics with equal probability. Afterwards, the probability of a word token to topic *t* is determined based on the probabilistic model (Schwarz, 2018).

To prepare the raw data for the LDA, in the preprocessing, we removed capital spelling and cleared non-alphanumerical characters (e.g., “.” “,” “;” “/” “-”) from the text strings to avoid interference with the clustering. The algorithm excluded stopwords (a list of highly frequent words that do not help with the classification of the documents, such as “and” and “or”) and all other short words (<4 characters) from the sampler. We used a predefined list of German stopwords and extended it by the following text-corpora-specific stopwords (English translations, original German words in brackets): accordingly [*entsprechend*]; with regard to [*hinsichtlich*]; in particular [*insbesondere*]; possibly [*gegebenenfalls*]; respectively [*beziehungsweise*]; eventual [*eventuell*]; child [*kind*]; relevant [*betroffen*]; evaluator [*sachverstaendige*]; question [*frage*]; perspective [*sicht*]; result in [*gibt; ergeben*]; as well as [*sowie*]; already [*bereits*]; further [*weiteren*].

Latent Dirichlet allocation makes it possible to estimate any number of topics. Literature indicates that there is no single best way or standard practice for determining the number of topics for LDA, and human judgments of the interpretability of topics have to be taken into account (Röder et al., 2015). A theoretical approach to determine the number of topics may lead to the authors of a study simply choosing the number of topics. In our study, based on theoretical assumptions, we expected a number of *T* = 4 topics to best represent our data, because these included cases relating to (a) child custody; (b) visitation; (c) child custody and visitation; and (d) child endangerment/child protection.

In recent years, several methods have been developed to provide empirical evidence for the number of chosen topics. Two of these are model perplexity (e.g., Griffiths and Steyvers, 2004) and topic coherence (e.g., Chang et al., 2009; Newman et al., 2010; Lau et al., 2014; Lau and Baldwin, 2016). Model perplexity (predictive likelihood) is a standard measure of performance for statistical models of natural language. Perplexity indicates the uncertainty in predicting a single word with chance performance resulting in a perplexity equal to the size of the vocabulary (Griffiths and Steyvers, 2004). Perplexity captures how surprised a model is by new data that it has not seen before, and it is measured as the normalized log-likelihood of a held-out test set. However, recent studies have shown that perplexity and human judgment often do not correlate and even sometimes slightly anticorrelate. Optimizing for perplexity may therefore not yield humanly interpretable topics (Chang et al., 2009).

The literature indicates that humans judge topics to be more consistent based on word co-occurrences (Chang et al., 2009; Lau et al., 2014; Lau and Baldwin, 2016). Therefore, the literature suggests using coherence measures to offer empirical evidence for the number of chosen topics, because those are based on word co-occurrence in each topic and may outperform other measures (Röder et al., 2015). Several coherence confirmation measures exist. In this study, we applied UMass coherence (Mimno et al., 2011), which is an asymmetrical confirmation measure between top word pairs (smoothed conditional probability) that account for the ordering among the top words (the most probable words) of a topic.

**Figure 1:**
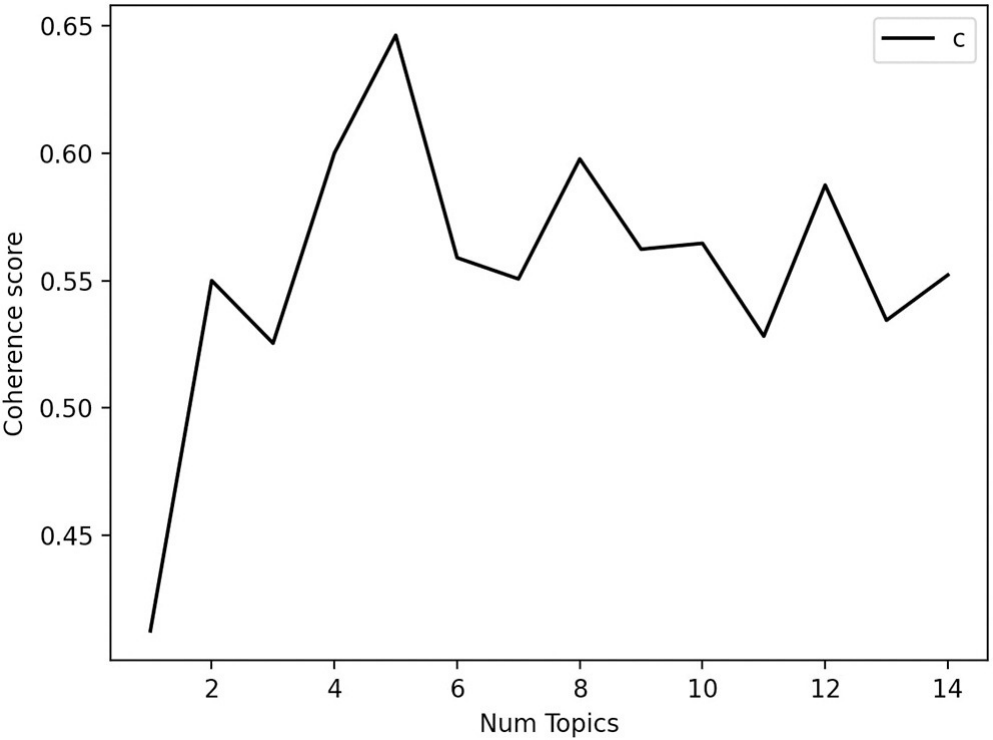
Coherence scores for numbers of topics. Num topics = 4 has a coherence value of 0.6000; Num topics = 5 has a coherence value of 0.6463; Num topics = 6 has a coherence value of 0.5589.

Figure 1 displays the results of the coherence scores for up to 15 topics. In our case, the number of five topics revealed the highest coherence score. Given the expected number of four topics based on theoretical appraisal, we compared the interpretability of the LDA models for four and five topics. Taking both the higher coherence score and the clearer interpretability of the model with five topics into account, we concluded that *T* = 5 topics best fitted our data and computed our LDA accordingly.

Because Gibbs sampling is a Bayesian technique, it requires a prior probability distribution of the hyperparameters α and β. Based on the number of topics *T* = *5*, we chose α = 0.25; and based on the size of the vocabulary *V* = 1194, we chose β = 0.1 (see Griffiths and Steyvers, 2004). In a burn-in of 500 iterations, we sampled new topic assignments for all words excluding stopwords. When taking this approach, the Markov chain converges toward the maximum of the likelihood function. After the burn-in period, we took 10 samples from the Gibbs sampler to obtain an approximation of the parameter values. To guarantee the statistical independence of the samples, we ran 50 additional iterations of the sampling process in between the samples (see Schwarz, 2018). All analyses were computed using PYTHON 3. PYTHON's built-in *Gensim* package uses a variational Bayes sampling method that is described as faster but less precise than Mallet's Gibbs sampling. We therefore made use of a wrapper to implement the *Mallet* (Machine Learning for Language Toolkit) package from within PYTHON's built-in *Gensim* package, to run our analysis based on Gibbs sampling.

We further used PYTHON's pyLDAvis, a visualization tool that supports graphical plotting of LDA results. PyLDAvis is a chart that plots the topics as clusters with two principal components and shows the proportion of the most probable words in each topic (also referred to as top words). Marginal distributions of the five topics are displayed on the left-hand side of the plots (the larger the circle, the more prevalent that topic is within the data). A good topic model will show fairly big, non-overlapping circles scattered throughout the chart instead of being clustered in one quadrant. Overall frequencies for all top words and estimated frequencies of top words within the topic are displayed on the right-hand side of the plots.

As a final step, we used probability scores to identify the 15 most probable keywords for each topic from an LDA for each topic. We then manually coded these keywords as (1) legal term (e.g., custody, physical custody, termination, and child's best interest); (2) forensic issue/psychological construct (e.g., relationship, attachment, parenting ability/capacity, stability, impairments, and disability); or (3) other (e.g., parent, contact, mother, and household). Two independent coders (one psychological and one legal professional) conducted the coding. The kappa score for the interrater reliability was 0.93. In case of conflicting coding, a third coder was consulted.

## Results

### Latent Dirichlet Allocation Model

The word probability matrix was created for a total vocabulary size of *V* = 1,194 words. We utilized the LDA model to analyze the latent topic structure across documents and to identify the most probable words (top words) within topics.

Table 1 displays the topics, the assigned labels, and the example text string with the highest probability for each topic (English translations). Regarding their content, we labeled the topics “child endangerment, child protection”; “custody, residence”; “visitation and custody, residence”; “child endangerment, parenting capacity,” and “visitation.” Text strings with the highest probability for each topic as displayed in Table 1 consist of *min* = 54 up to *max* = 192 words.

**Table 1:** Topics, dimensions, and example text strings with the highest probabilities.

**Topic**	**Dimension**	**Example text string**	***p***
1	Child endangerment, child protection	Are the child's parents willing and able to ensure the care and upbringing of the child, taking into account any special requirements of the child and, if necessary, to put aside their own interests? Has damage to the child already occurred or is there already a danger at present to such an extent that considerable damage to the child's development can be foreseen with a reasonable degree of certainty? How serious and probable are the threatening impairments of the child? Are there other measures of help and support to avert the endangerment than a termination of parental custody or parts of it, if yes which ones? Are the parents willing and able to accept and implement these measures so that the endangerment is averted? What are the child's ideas about the relationship with her or his parents and how are they to be weighed in terms of goal orientation, intensity, stability, and autonomy?	0.9945
2	Custody, residence	With which parent should the child be living, taking into account the best interests of the child? Can it be expected that the abolition of joint custody would serve the child's interests best? The expert is requested to also work toward establishing an agreement between the parties involved when conducting the evaluation.	0.9970
3	Visitation and custody, residence	To what extent are both parents prepared and able to provide for the care and upbringing of the child, taking into account any special individual requirements of the child and, if necessary, to set aside their own interests? How is the requirement of a uniform and stable upbringing to be weighed in educational, personal, and local respects? What are the nature and intensity of the child's emotional attachment to the parents and the relationships with siblings and close caregivers? How are the child's expressions of its preference to be weighed in terms of goal orientation, intensity, stability, and autonomy? Are the parents able and willing to maintain and promote the personal contact of the child with the other parent and to have a supporting effect on the child in this sense? Are there indications that the parents might be in a position to jointly exercise child custody, in particular, the physical custody and custody in school matters, following the best interests of the child principle? If so, under what conditions could this be achieved? If not, which arrangements best meet the child's best interests, taking into account the general criteria of the child's best interest?	0.9932
4	Child endangerment, parenting capacity	Whether the child's well-being would be endangered if the child remained in the household of the child's parents? The evaluation is also to regard the following questions: Is the mother of the child alone or in domestic community with the father of the child suitable and able to raise and care for her son? Is the father of the child alone or in domestic community with the mother of the child suitable and able to raise and care for his son? In the case of restrictions in the parenting capacity of the mother or the father of the child, can a threat to the child's well-being also be countered sufficiently by outpatient measures, the use of family assistance, or other support services?	0.9952
5	Visitation	What relationship does the child have to the parent who seeks visitation? What is the quality of the child's relationship with both parents? What form of visitation/contact is in line with the voice of the child? Do the greater disadvantages arise from obeying or ignoring the voice of the child? Are there any indications of disadvantageous behavior of one parent that affects the child's attitude toward visitation/contact? Are the parents seeking visitation able to arrange the contact in a child-friendly manner? What possibilities are there to make the contact or the handover situation favorable and less stressful for the child? Is there a danger of physical or psychological damage to the child through the visitation/contact? Can this danger be averted by supervised contact or by other measures; if necessary, by which ones?	0.9946

*English translations of the example text strings are displayed*.

To further present aggregated information on the content of the forensic assignments, Table 2 displays the topics, labels, and each top 15 words with probabilities. We present English translations of the words (original German words in brackets). Results indicate that a few terms such as “child's best interest” or “ability/capacity” were among the most probable words in various topics. However, most of the terms occurred only among the most probable words within one topic.

**Table 2:** Topics, dimensions, and keywords with the highest probability.

**Topic**	**Dimension**	**Fifteen most probable keywords**	**Probability (*p*)**
1	Child endangerment, Child protection	Endangerment [*Gefaehrdung*]	0.035
		Harm [*Schaedigung*]	0.034
		Exist [*besteht*]	0.030
		Danger [*Gefahr*]	0.030
		Mother [*Kindesmutter*]	0.024
		Avert [*abzuwenden*]	0.023
		Refrain from [*laesst*]	0.019
		Impairments/disabilities [*Beeintraechtigungen*]	0.019
		Support services [*Unterstuetzungsangebote*]	0.019
		Certainty [*Sicherheit*]	0.018
		Already [*bereits*]	0.017
		Occurred [*eingetreten*]	0.017
		Substantial [*erhebliche*]	0.017
		Fit/suited [*geeignet*]	0.017
		Implement [*umzusetzen*]	0.016
2	Custody, residence	Exist [*besteht*]	0.026
		Capacity [*Faehigkeit*]	0.025
		Willingness [*Bereitschaft*]	0.024
		Intensity [*Intensitaet*]	0.024
		Parents [*Eltern*]	0.023
		Stability [*Stabilitaet*]	0.021
		Individual [*eigene*]	0.019
		Consideration [*Beruecksichtigung*]	0.019
		Requirements [*Anforderungen*]	0.018
		Safeguard [*gewaehrleisten*]	0.018
		Potentially [*etwaiger*]	0.017
		Education/parenting [*Erziehung*]	0.017
		Interests [*Belange*]	0.016
		Custody [*Sorge*]	0.016
		Goal orientation [*Zielorientierung*]	0.014
3	Visitation and custody, residence	Best [*besten*]	0.045
		In accordance with [*entspricht*]	0.044
		Interest [*Wohl*]	0.042
		Child's best interest [*Kindeswohl*]	0.029
		Residence [*Lebensmittelpunkt*]	0.022
		Child's best interests [*Kindeswohls*]	0.020
		Consideration [*Beruecksichtigung*]	0.020
		Mother [*Kindesmutter*]	0.018
		Visitation arrangement [*Umgangsregelung*]	0.017
		Parent [*Elternteil*]	0.014
		Visitation [*Umgang*]	0.012
		Joint [*gemeinsamen*]	0.011
		Mother [*Mutter*]	0.011
		Termination [*Aufhebung*]	0.010
		Father [*Kindesvater*]	0.010
4	Child endangerment, parenting capacity	Mother [*Kindesmutter*]	0.045
		Parenting capacity [*Erziehungsfaehigkeit*]	0.036
		Parents [*Kindeseltern*]	0.032
		Ability/capacity [*Lage*]	0.031
		Household [*Haushalt*]	0.027
		Educate/to parent [*erziehen*]	0.025
		Endangered [*gefaehrdet*]	0.016
		Child maltreatment/endangerment [*Kindeswohlgefaehrdung*]	0.015
		Fit/suited [*geeignet*]	0.014
		Father [*Kindesvater*]	0.013
		Endangerment [*Gefaehrdung*]	0.013
		Give care/to parent [*betreuen*]	0.013
		Case [*Falle*]	0.012
		Support [*foerdern*]	0.012
		Child's best interests [*Kindeswohls*]	0.011
5	Visitation	Visitation [*Umgang*]	0.081
		Parent [*Elternteil*]	0.029
		Ability/capacity [*Lage*]	0.026
		Personal contact [*Umgangskontakte*]	0.019
		Create [*gestalten*]	0.018
		Endangerment [*Gefahr*]	0.018
		Relationship/attachment [*Beziehung*]	0.017
		Measures [*Maßnahmen*]	0.016
		Physical [*koerperlichen*]	0.015
		Child's best interest [*Kindeswohl*]	0.015
		Psychological/mental [*seelischen*]	0.014
		Meets [*entspricht*]	0.014
		Impairments [*Schaeden*]	0.013
		Seeking [*begehrenden*]	0.012
		Parents [*Elternteilen*]	0.012

*English translations of the most probable words presented; original German words in brackets*.

**Figure 2:**
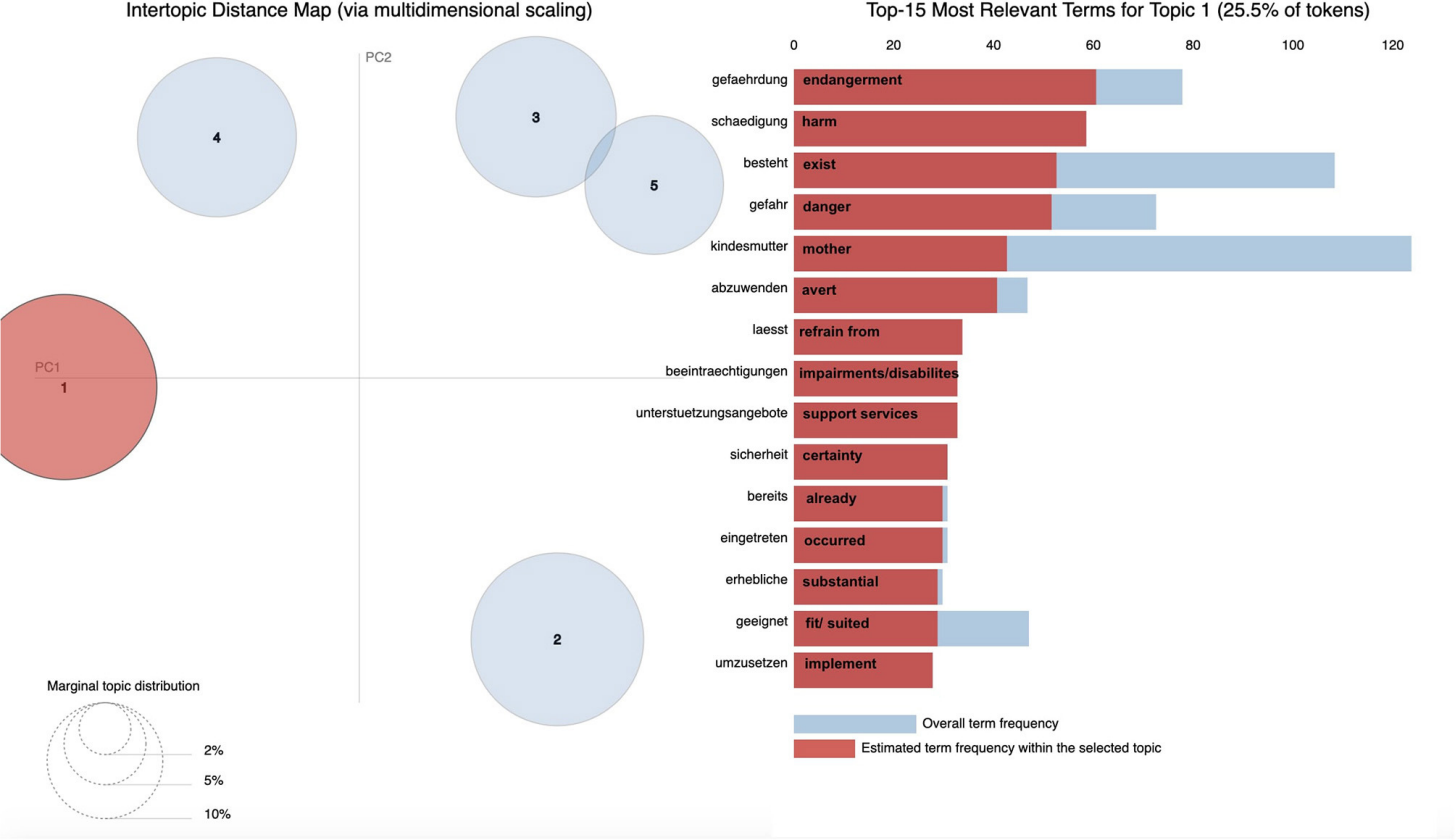
Marginal distributions of topics and estimated frequencies for the most probable words within Topic 1 “child endangerment, child protection”.

**Figure 3:**
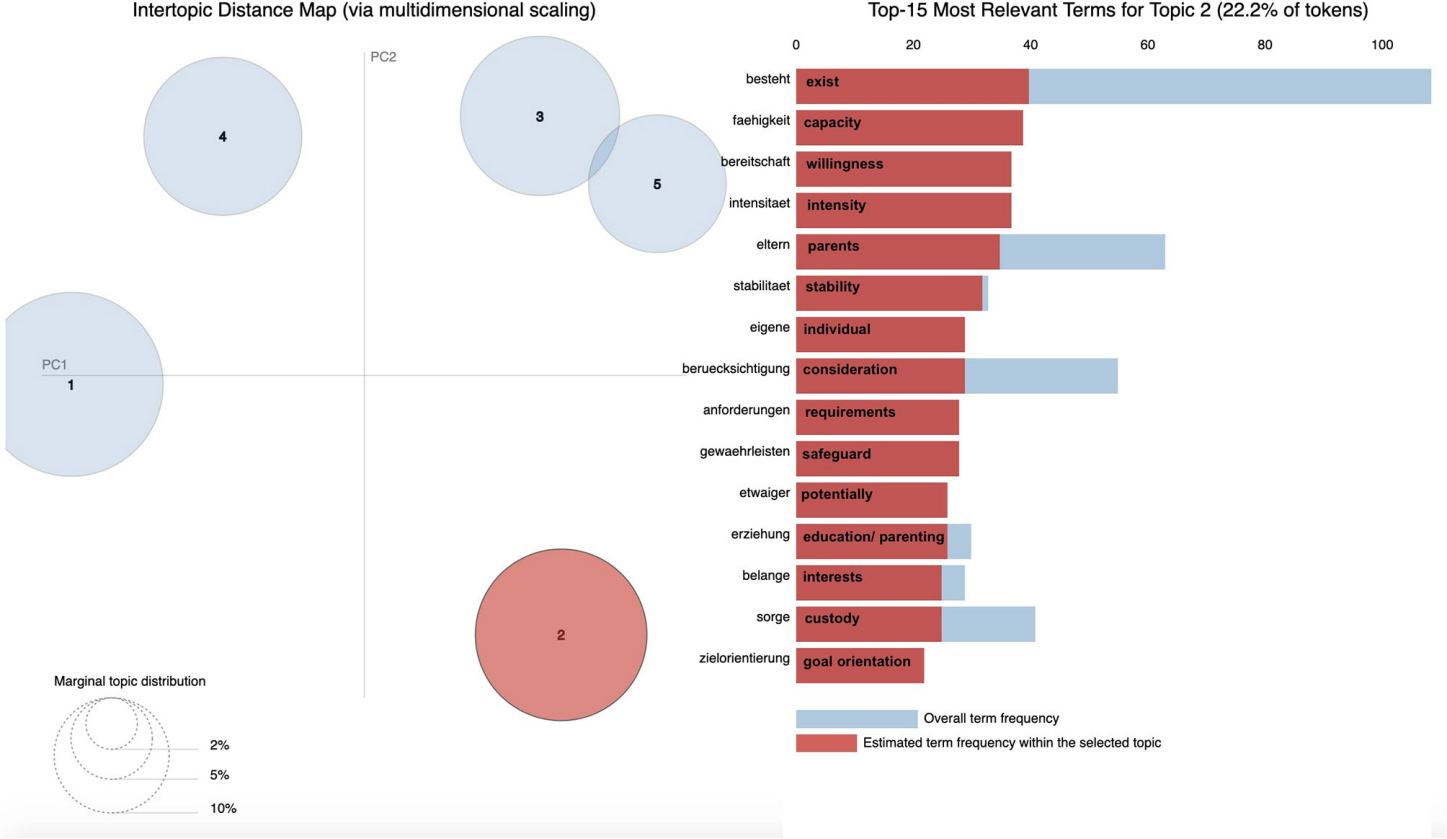
Marginal distributions of topics and estimated frequencies for the most probable words within Topic 2 “custody, residence”.

**Figure 4:**
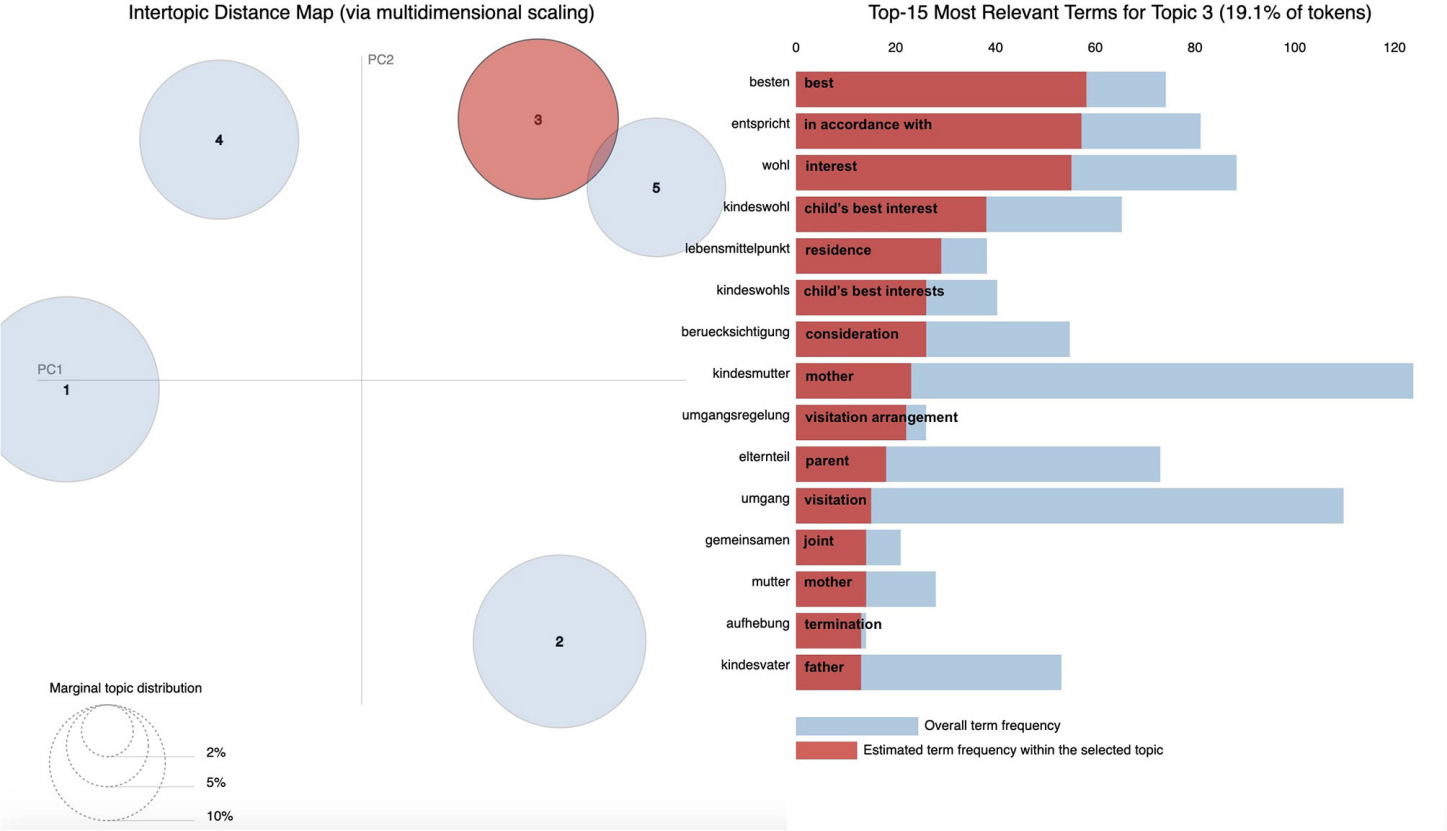
Marginal distributions of topics and estimated frequencies for the most probable words within Topic 3 “visitation and custody, residence”.

**Figure 5:**
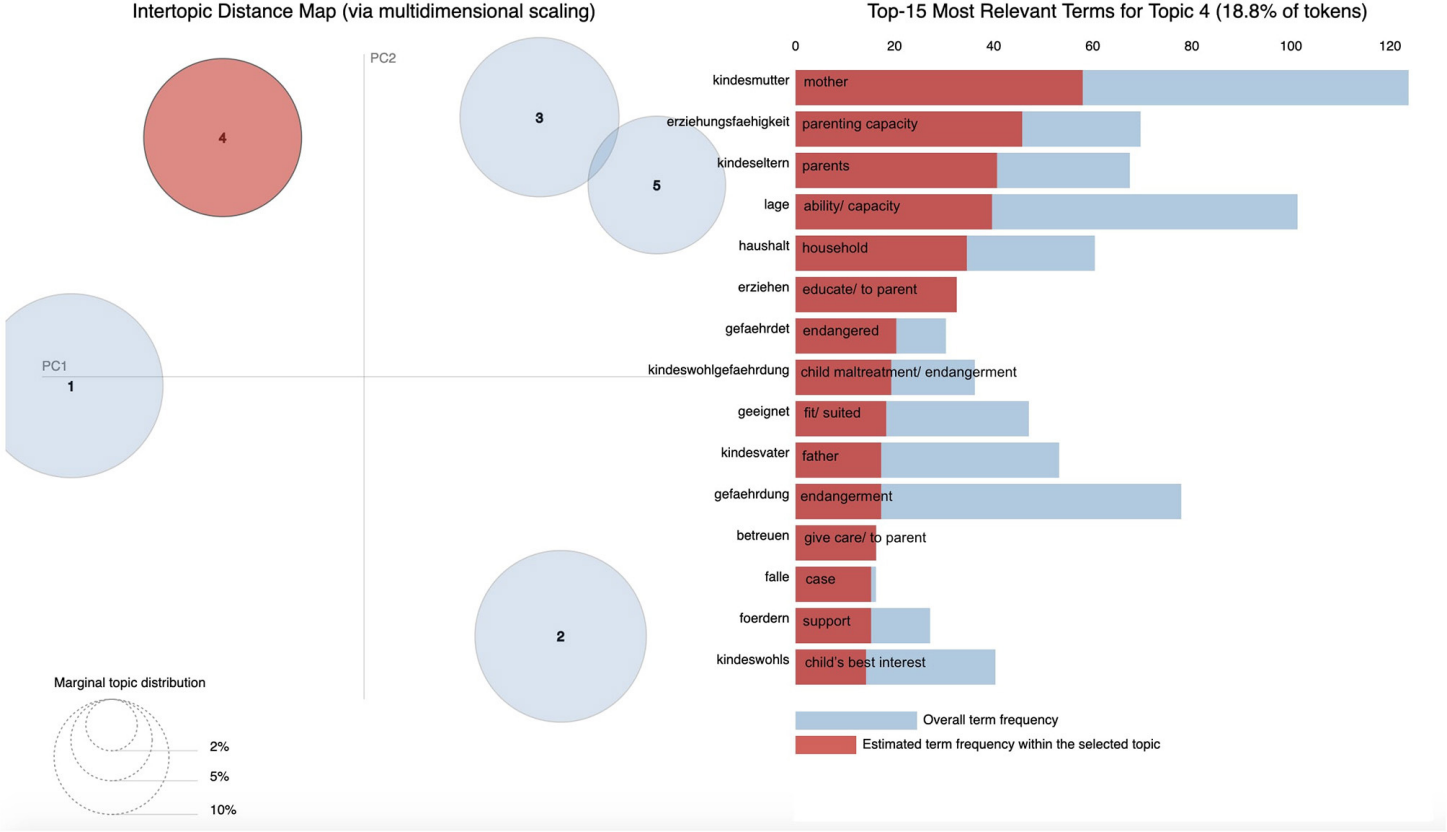
Marginal distributions of topics and estimated frequencies for the most probable words within Topic 4 “child endangerment, parenting capacity”.

**Figure 6:**
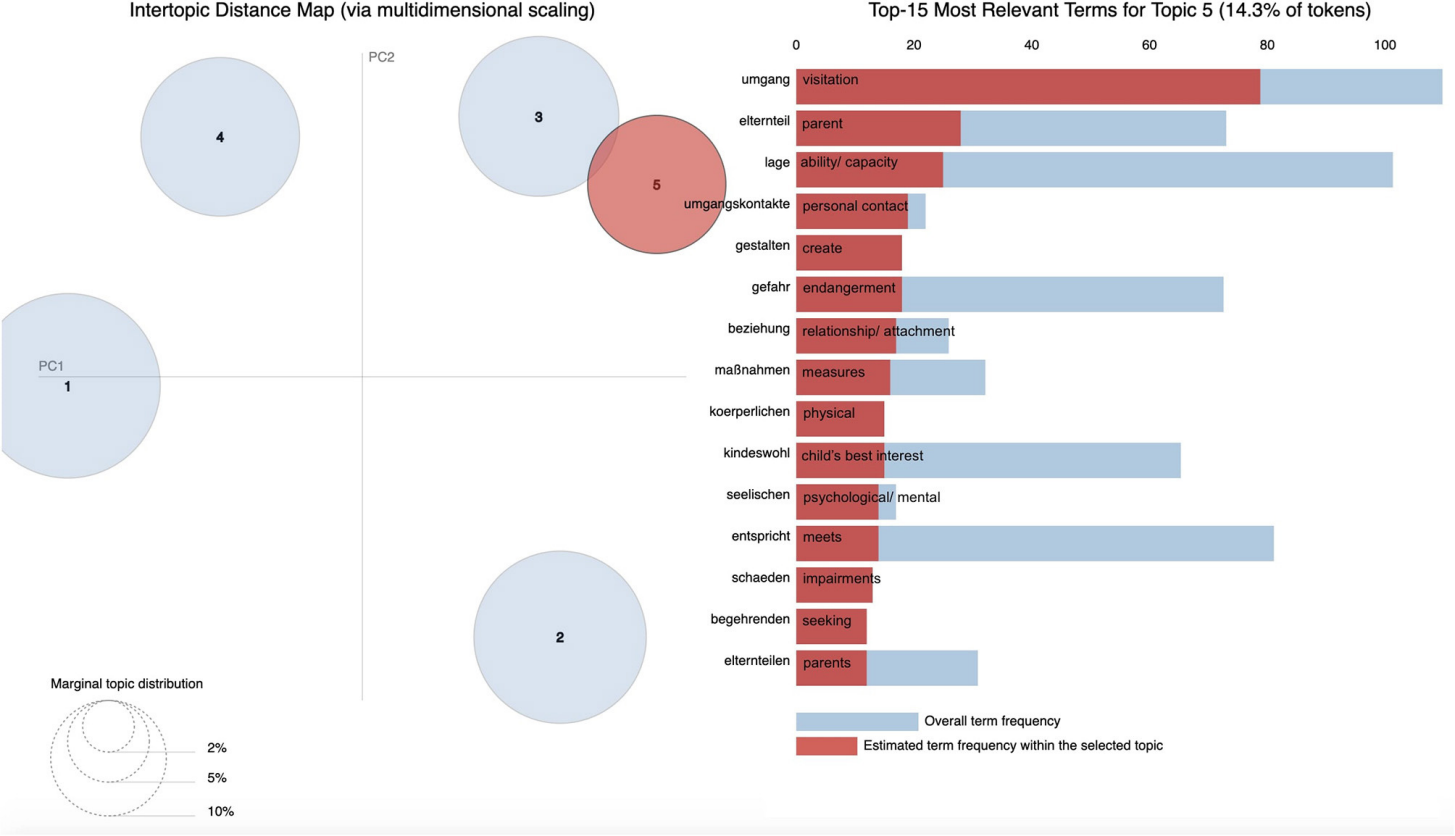
Marginal distributions of topics and estimated frequencies for the most probable words within Topic 5 “visitation”.

With the use of pyLDAvis, Figures 2–6 display graphic representations of the LDA results. Visualization of the marginal distributions of the five topics showed that Topics 1 (“child endangerment, child protection”), 2 (“custody, residence”), and 4 (“child endangerment, parenting capacity”) do not overlap. We found a slight overlap between Topics 3 (“visitation and custody, residence”) and 5 (“visitation”). Overall frequencies and estimated frequencies for top words within each topic are displayed on the right-hand side of the plots (note that we plotted the original German words; we have added English translations for each word to the plots). Visualization of our topic model overall supported our conclusion to compute our final topic model for a number of five topics.

### Coding of the Most Probable Words and Final Topic Model

**Figure 7:**
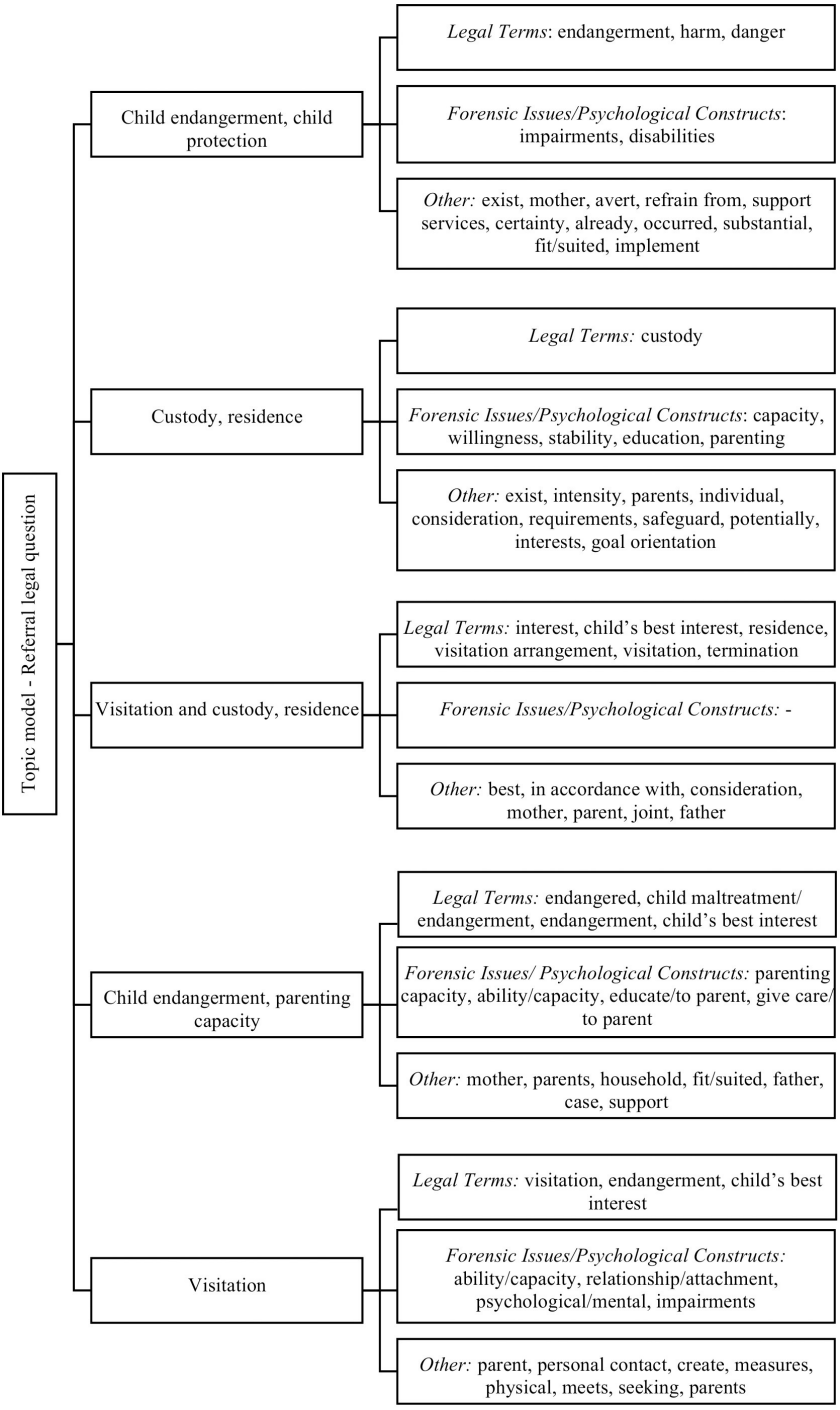
Final topic model based on coding of the most probable words.

To quantify the content of the most probable keywords presented in Table 2, we then coded the most probable 75 words across topics as (1) legal term, (2) forensic issue/psychological construct, or (3) other. The coders' appraisal of the most probable 75 words is displayed in Figure 7 that presents the final topic model.

For all but one topic, the most probable 15 words included both legal terms and forensic issues/psychological constructs as well as other terms. Within Topics 1 (“child endangerment, child protection”) and 5 (“visitation”), legal terms occurred with higher probabilities than forensic issues. Within Topics 2 (“custody, residence”) and 4 (“child endangerment, parenting capacity”), forensic issues occurred with higher probabilities than legal terms. The most probable 15 words for the topic “visitation and custody, residence” comprised only legal and other terms.

Legal terms among the most probable words varied depending on the underlying topics. This indicated that the forensic assignments contained case-specific legal terms such as “visitation,” “residence,” “custody,” or “child endangerment.” Forensic issues/psychological constructs also varied depending on the underlying topics. In cases of child custody and visitation, they included constructs such as “capacity/ability,” “parenting,” “relationship/attachment,” or “stability.” In questions on child endangerment and child protection, Topic 4 was characterized by the most probable words related to the psychological construct of parenting capacity, whereas Topic 1 was characterized by the most probable words related to the legal concept of child protection.

## Discussion

### Discussion of Main Findings

The translation of the referral *legal question* into case-specific psychological questions addressing *forensic issues* is a key variable in assuring accurate and effective forensic evaluations (American Psychological Association, 2013; LaDuke, 2017). The aim of this study was to clarify the identification and definition of the relevant constructs to be examined by forensic experts from the perspective of the courts. This helps to provide clarity on what is actually at stake within the questions posed by the courts and whether forensic psychologists can respond to this.

Our first research question concerned the *underlying topics* (dimensions) that can be identified in the referral questions posed by the judges. Based on theoretical assumptions, we expected a number of four topics to best represent our data, because they consisted of cases relating to (a) child custody, (b) visitation, (c) child custody and visitation, and (d) child endangerment/child protection. Empirically, we found that five topics revealed the highest coherence score. Taking both the higher coherence score and the clearer interpretability of the model with five topics into account, we concluded that *T* = 5 topics best fits our data (which was supported by the visualization of our results). Accordingly, we found not one but two topics representing cases on child endangerment.

The LDA analysis identified five primary topics in referrals to which we assigned the following labels: “visitation”; “visitation and custody, residence”; “custody, residence”; “child endangerment, parenting capacity”; and “child endangerment, child protection.” Our results on the probability distributions of the most probable words within the five topics indicate that questions on child endangerment are involved in three out of five topics. Interpreting our findings on the most probable words, the five topics resemble five case constellations in which questions address the following:

a) potential child endangerment in the context of visitation contacts (Topic 5),
b) a possibly limited parenting capacity and its potential effects on child well-being (Topic 4),
c) an impairment/endangerment of the child has already occurred or will potentially occur (Topic 1).

Furthermore, we found two topics concerning the custody and residence of the child in which questions address the following:

d) a better option concerning custody and residence (Topic 2), and
e) a topic that cannot be defined clearly that deals with questions on custody, residence, and visitation in which the most probable words indicate no questions on specific psychological constructs (Topic 3).

Our second research question concerned probability *distributions of legal terms and forensic issues/psychological constructs within the referral questions* posed by family law judges. For all five topics, the 15 most probable keywords include legal terms. For four out of five topics, the most probable words include forensic issues. Topics 1 (“child endangerment, child protection”) and 5 (“visitation”) are characterized by a stronger focus on legal concepts within the referral questions. Within Topics 2 (“custody, residence”) and 4 (“child endangerment, parenting capacity”), assignments are oriented more toward psychological constructs. Within Topic 3 (“visitation and custody, residence”), no forensic issues/psychological constructs occur among the most probable words.

These findings may lead to the interpretation that in four of the five topics that we identified, the judges utilize their referral questions to define the legal framing, as well as to ask for case-group-specific psychological information. This refers to the three topics in which questions on child endangerment are involved, as well as to the topic addressing which is the better option concerning custody and residence. With regard to these cases, it seems quite clear in which areas forensic evaluators can contribute their specific expertise.

However, in one topic that deals with questions on custody, residence, and visitation, judges seem to struggle to define criteria that forensic evaluators should assess. We find a slight overlap between Topics 3 (“visitation and custody, residence”) and 5 (“visitation”), but in contrast to Topic 5, questions on a potential endangerment of the child do not seem to be included in Topic 3. It seems that in some cases dealing with visitation, custody, and residence of the child after a parental separation, it is rather unclear from the judge's perspective what empirical-psychological knowledge should contribute to preparing a legal decision. From a psychological point of view, resolving the parental conflict might be most likely to be in the best interest of the child. However, in many cases, this cannot be achieved either by a court order on child custody or visitation rights or by an expert evaluation that is of a diagnostic nature. Instead of assigning an expert evaluation, it might therefore be more beneficial to assign intervention measures such as mediation, with the goal of reducing the parental conflict.

Across the topics described above, we found only a few psychological constructs among the most probable words in various topics (e.g., “child's best interest” and “ability/capacity”). Most of the psychological constructs occur only among the most probable words within one topic, indicating that the psychological constructs at question vary according to the underlying case structure. This finding is in line with theoretical expectations, because the variety of legal and forensic issues that may potentially be assessed in family law proceedings is quite broad and case-specific, with the child's best interest principle being the only overarching criterion (Miller, 2002; American Psychological Association, 2010, 2013).

Overall, our data support the conclusion that specifying the best interest criterion from a judge's standpoint does not lead to the identification of a general set of factors (or a general set of questions) that are to be assessed. Factors vary with the underlying case structure. Example text strings with the highest probabilities within topics do not appear as general orders or simply name judicial statutes (see Gould, 1999; Coester, 2016). Example text strings vary in length and wording and appear to be tailored to the individual case.

### Limitations

Our study has several limitations. First, it includes only data from an *ad hoc* sample in Northern Germany, and results cannot be generalized. Second, we cover only a 2-year timeframe in our sample and are therefore unable to analyze time trends. Third, due to anonymization, we are unable to control for preferences in the wording of specific judges. It cannot be ruled out that this had an effect on probabilities in wording within the sample. Fourth, it needs to be stated that LDA analysis is a rather new technique and that there is not yet any uniform mechanism to evaluate topic models based on LDA analysis (see Chang et al., 2009; Newman et al., 2010; Lau et al., 2014; Röder et al., 2015; Lau and Baldwin, 2016). Therefore, it is important to note that our results were generated in an explorative approach, and replications are still pending. Nonetheless, our analysis does deliver a first step toward a systematic investigation of the translation process from legal questions to forensic issues in child custody evaluations.

### Open Questions for Future Research

In this study, LDA proves to be a suitable method with which to discover topics and identify latent structures by analyzing raw data derived from court files. This opens up a broad range of possible further research questions to be pursued with this technique. Latent Dirichlet allocation techniques have the potential to include a broader content of court files such as the expert's recommendations and the court's decisions. Therefore, future research could, for example, focus on whether the forensic experts give sufficient answers to the referral questions.

Future research could also include Delphi studies with judges on the one side and forensic evaluators on the other. This approach could aim to determine which information both sides consider relevant to clarify the forensic-psychological issues that need to be assessed as prerequisites for assessing the legal issues.

### Prospects for Practical Implementation

In both civil and common law systems, expert opinions should cover “issues of fact” and may not offer “legal” conclusions (see World Bank, 2010). The final and overall responsibility for answering the legal question lies with the court (see Bergmann, 2018). Experts are called to provide data and expertise on a number of forensic issues. However, which forensic issues are deemed relevant might determine to a significant degree which legal decisions are made. The process of determining relevant forensic issues is therefore of great practical importance.

On the basis of this exploratory study, it is certainly too early to give comprehensive recommendations for practice. However, our analysis does deliver a first step toward a systematic investigation of the translation process from legal questions to forensic issues in child custody evaluations. We tentatively conclude that a mutual basic understanding of the legal and psychological foundations would seem to be crucial. Forensic evaluators must understand the meaning of legal statutes. Only then can they understand what is the objective and aim of the judicial proceeding in general and the forensic evaluation in particular. Unclear or erroneous referral questions posed by the judge must be pointed out, and clarification must be requested. The judge, on the other hand, must have a basic understanding of psychological concepts relating to the best interests of the child principle, because the courts are required to examine a forensic evaluation for consistency and comprehensibility and to ensure that fundamental general professional standards are observed (Specialty Guidelines for Forensic Psychologists: American Psychological Association, 2012). In case of doubt, mutual clarification processes must be initiated at an early stage.

Thus, in the process of translating from legal questions to forensic issues, it may be advisable for forensic evaluators to first identify the constructs named by the judges within their referral questions and, in a second step, to fill them with assessable empirical indicators. For example, if the court's question is “Is the parenting capacity so limited that the child is endangered?,” the forensic evaluator would have to specify indicators (and in a next step instruments) that make the constructs “parenting capacity” and the “child's endangerment” assessable from a psychological perspective. The indicators specified by the forensic evaluators can, in a third step, be presented to the judge and, if necessary, adapted in consultation with that judge. The overarching aim is to ensure that those forensic issues are identified whose evaluation will contribute to answering the legal questions appropriately.

## Data Availability Statement

The raw data supporting the conclusions of this article will be made available by the authors, without undue reservation.

## Ethics Statement

Ethical review and approval was not required for the study on human participants in accordance with the local legislation and institutional requirements. Written informed consent for participation was not required for this study in accordance with the national legislation and the institutional requirements.

## Author Contributions

All authors listed have made a substantial, direct and intellectual contribution to the work, and approved it for publication.

## Conflict of Interest

The authors declare that the research was conducted in the absence of any commercial or financial relationships that could be construed as a potential conflict of interest.
